# Particle beam radiation therapy for sinonasal malignancies: Single institutional experience at the Shanghai Proton and Heavy Ion Center

**DOI:** 10.1002/cam4.3393

**Published:** 2020-09-25

**Authors:** Weixu Hu, Jiyi Hu, Qingting Huang, Jing Gao, Jing Yang, Xianxin Qiu, Lin Kong, Jiade J. Lu

**Affiliations:** ^1^ Department of Radiation Oncology Shanghai Proton and Heavy Ion Center Shanghai China; ^2^ Shanghai Engineering Research Center of Proton and Heavy Ion Radiation Therapy Shanghai China; ^3^ Department of Radiation Oncology Shanghai Proton and Heavy Ion Center Fudan University Cancer Hospital Shanghai China

**Keywords:** carbon‐ion radiotherapy, nasal and paranasal sinus malignancies, particle‐beam radiation therapy, proton radiotherapy, sinonasal malignancies

## Abstract

**Background:**

Sinonasal malignancies (SNM) include malignant neoplasms of various histologies that originate from the paranasal sinuses or nasal cavity. This study reported the safety and efficacy of particle‐beam radiation therapy (PBRT) for the treatment of sinonasal malignancies.

**Methods and materials:**

One‐hundred‐and‐eleven patients with nonmetastatic sinonasal malignancies received definitive (82.9%) or salvage (31.5%) PBRT. The majority (85.6%) of patients presented with T3/4 disease, and only 19 (17.1%) had R0 or R1 resection. Seventy (63.1%) patients received carbon‐ion radiotherapy (CIRT), 37 received proton radiotherapy (PRT) followed by CIRT boost, and 4 received PRT alone. Prognostic factors were analyzed using Cox regression for univariate and multiple regression. Toxicities were reported using the Common Terminology Criteria for Adverse Events (version 4.03).

**Results:**

The median follow‐up was 20.2 months for the entire cohort. The 2‐year local progression‐free survival (LPFS), regional progression‐free survival (RPFS), distant metastasis‐free survival (DMFS), progression‐free survival (PFS), and overall survival (OS) rates were 83%, 97.2%, 85.9%, 66%, and 82%, respectively. Re‐irradiation and large GTV were the significant factors for OS. Melanoma and sarcoma patients had significantly higher distant metastatic rate, and poorer OS and PFS. Late toxicity occurred in 22 (19.8%) patients, but only 4 (3.6%) patients experienced grades 3‐4 late toxicity.

**Conclusions:**

Particle‐beam radiation therapy results in excellent local‐regional control with extremely low serve toxicities for patients with SNM. Sarcoma and melanoma were featured with a greater risk of death from distant dissemination. Patients who underwent re‐irradiation had significantly worse OS. PBRT is feasible and safe in the management of SNM.

## Graphical abstract

PBRT achieved excellent local‐regional control with extremely low serve toxicities in the management of sinonasal malignancies (SNM). PBRT is an effective and safe treatment option for patients with SNM.

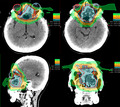

## 1 INTRODUCTION

Sinonasal malignancies (SNM) include malignant neoplasms of various histologies that originate from the paranasal sinuses or nasal cavity. It is a relatively rare condition and accounts for 3%~5% of head and neck malignancies.^1^ Despite of its rarity, SNM possess a major treatment challenge for two reasons. It is usually diagnosed in advanced stage due to its inconspicuous location and late occurrence of symptoms.^2^, ^3^ Complete surgical resection with sufficient margins is often not feasible because of the vital structures adjacent to the tumor and the extensive stages at diagnosis. Radiation therapy (RT) plays an important role in the management of SNM patients, either adjuvantly or definitively.^4^ However, dose escalation of photon‐based RT including the more advanced intensity‐modulated radiotherapy (IMRT) for patients with gross lesion(s) is limited by the nearby critical organs at risk (OARs). The efficacy of IMRT over conventional RT for disease control is controversial has not been confirmed, although IMRT could substantially reduce radiation‐induced adverse events.^5^, ^6^, ^7^, ^8^, ^9^ Clearly, more effective treatment is needed especially for patients with local or regionally advanced disease.

Particle‐beam radiation therapy (PBRT), typically using accelerated proton or carbon‐ion, has the advantage of small penumbra and a dose‐focusing Bragg peak followed by a rapid fall‐off thereby producing a more superior dose distribution as compared to photon‐based IMRT.^10^, ^11^, ^12^ Furthermore, carbon‐ion beam has higher linear energy transfer (LET) and relative biological effectiveness (RBE) which enables more effective cell killing through inducing more DNA double‐bond damage. The higher RBE of heavy particles is important in the treatment of radioresistant malignancies such as melanoma, sarcoma, and adenoid cystic carcinoma (ACC). PBRT has been proven technology in the treatment of head and neck tumors with complex anatomic features. The published outcomes have suggested potential advantages of PBRT in both disease control and toxicity profiles for patients with SNM.^12^, ^13^ However, most studies were of retrospective nature with relatively small sample size, and multi‐institutional case series or meta‐analysis usually suffer from heterogenous and nonstandardized clinical practice.^14^, ^15^ With this background, the aim of this study is to bolster the current literature by documenting the outcomes of a relatively large group of patients with SNM treated with PBRT at the Shanghai Proton and Heavy Ion Center.

## 2 METHODS AND MATERIALS

### 2.1 Eligibility

This retrospective was approved by the institutional review board (IRB) of the Shanghai Proton and Heavy Ion Center (SPHIC) (ethical code: 200220EXP‐02). All patients obtained written informed consent before enrolling in this study. The inclusion criteria are as follows: (a) newly diagnosed cases were confirmed by pathology, (b) pathological and/or radiological diagnoses were obtained for patients presented for salvage therapy, (c) no distant metastasis at diagnosis, and (d) the Eastern Cooperative Oncology Group (ECOG) performance status of 0 to 2. One‐hundred‐and‐eleven patients were enrolled in this study.

### 2.2 Pretreatment evaluation

The diagnosis of all patients presented for definitive treatment was histologically confirmed. Pathological and/or radiological diagnoses were obtained for patients presented for salvage therapy. Pretreatment evaluations included a complete history and physical examination (H&P), full blood count (FBC), hepatic/renal function tests, serum electrolytes, urine analysis, electrocardiogram, and MRI or CT (if MRI was contraindicated) of the head and neck region. FDG‐PET/CT was used to rule out distant metastasis but could be replaced by total body bone scan, enhanced abdominal CT or ultrasound, and enhanced CT of the thorax.

The 7th or 8th edition of the American Joint Committee for Cancer Staging System (depend on the date of diagnosis) was used to stage for all patients. All patients were thoroughly discussed in the multidisciplinary tumor clinic at SPHIC to confirm the indication of particle radiotherapy prior to their inclusion into our institutional cancer registry and treatment planning. The current study was approved by the institutional review board of SPHIC (ethical code: 200220EXP‐02) with a waiver of informed consent.

### 2.3 Particle‐beam radiation therapy

All patients were immobilized in supine position and immobilized using AlphaCradle^®^ (composite foamer without magnetism) and thermoplastic masks. CT without intravenous contrast at 1.5‐mm slice thickness was performed from the vertex to the inferior margin of clavicular heads for the purpose of simulation. MRI‐CT fusion was performed for the purpose of delineation. The GTV(s) including all disease foci discovered on clinical examination or imaging studies for patients with incomplete surgical resection in primary site (GTVp) or positive lymph nodes (GTVnd). The CTV(s) covering both primary site (CTV‐G) and of positive neck lymph nodes (CTV‐N) plus 1‐3 mm margin (depend on the proximity to OARs). CTV 1 included the pretreatment tumor bed (after R1 resection or achieved complete response [CR] after chemotherapy) and high‐risk areas for tumor extension; CTV 2 included the ipsilateral or bilateral jugular lymph node region, depending on cervical lymph node status and primary tumor extension. The planning target volume (PTV) was CTVs with a 3‐6 mm margin for range uncertainty with regard to dose distribution and potential setup errors. Proton radiotherapy (PRT) to 56 Gy (RBE) in 28 daily fractions or carbon‐ion radiotherapy (CIRT) to 56 Gy (RBE) in 28 daily fractions was used in patients who achieved R0 resection. A dose of 60‐66 Gy (RBE) in 30‐35 fractions using PRT, CIRT to 63‐73.5 Gy (RBE) in 18‐21 fractions, or a combination of PRT (54‐56 Gy (RBE)/27‐28 fractions) and a CIRT boost (15‐17.5 Gy (RBE)/5 fractions) was performed in patients with a gross tumor or after R1 resection. For re‐irradiation, CIRT using 63 Gy (RBE) in 21 fractions was given.

Doses of intensity‐modulated proton or carbon‐ion therapy (IMPT or IMCT) were prescribed in Gy (RBE). The RBE value for proton radiotherapy was 1.1 and for carbon‐ion radiotherapy was between 2.8 and 3.7 (depending on the depth in the spread‐out Bragg peaks). Dose constraints of the critical OARs of the head and neck were based on TD5/5 described by Emami et al,^16^ except for the optic nerve/chiasm and the temporal lobes, of which the experience from the National Institute of Radiation and Quantum Science (NIRQS), Japan, was used (D20 < 30GyE for optic nerve and chiasm; V40 < 7.66cc and V50 < 4.66cc for temporal lobes).^17^ All patients received pencil beam scanning (PBS)‐based IMPT and IMCT. Two or 3‐beam arrangements were typically used. The Siemens Syngo^®^ treatment planning system (TPS) was used for planning IMPT and IMCT. Weekly CT without IV contrast was performed for verifying dose distribution for all patients.

### 2.4 Surgery and systemic therapy

All patients were referred from head and neck surgeons or had consulted by a surgical oncologist. Surgery with an intention of complete resection was attempted for all patients based on the extent of the disease, patients' performance status, as well as preferences. Concurrent chemotherapy was performed in 19 RT‐naive patients including 2 patients with melanoma received temozolomide, and 17 patients with SCC, ONB, or poor‐differentiated carcinoma received platinum drugs. Systemic therapy was provided to patients based on the histology and stage of disease as well as the discretion of the medical oncologists.

### 2.5 Follow‐up

All patients were required to be followed‐up according to the Ethical Board approved standard follow‐up protocol of SPHIC after discharge. The first follow‐up was scheduled within 4‐6 weeks after the completion of particle radiotherapy. Patients would then be followed‐up every 3~4 months within first 2 years, every 6 months in the following 3 years, then annually thereafter. A complete H&P with a focus to head and neck area, FBC and serum electrolytes, MRI or CT scans (if MRI is contraindicated) of the head and neck regions are required by the protocol. Other studies such as thoracic and/or abdominal CT, abdominal ultrasound, and whole body PET‐CT can be performed based on the clinical indication.

### 2.6 Data analysis

The duration of overall survival was calculated from the time of diagnosis confirmation until death or last follow‐up. The time to local, regional, or distant failure was measured from the initiation of any treatment until disease progression or recurrence. Local progression‐free survival (LPFS), regional progression‐free survival (RPFS), distant metastasis‐free survival (DMFS), progression‐free survival (PFS), and overall survival (OS) rates were calculated using the Kaplan‐Meier method. COX regression model was used for both univariate and multivariate analyses of survival probabilities. All analyses were performed using the SPSS statistics package (Version 25.0).

Adverse events were defined and scored according to the CTCAE 4.03. Acute toxicities referred to adverse events occurred during or within 3 months after the start of PBRT. Late toxicity referred to those occurred after 3 months from or persisted for more than 3 months after the completion of PBRT.

## 3 RESULTS

### 3.1 Characteristics of patients

Between 5/2015 and 6/2019, 117 consecutive patients with SNM were treated at SPHIC. Six patients present with distant metastasis (DM) at diagnosis and treated palliatively were excluded from this analysis. The median follow‐up of the remaining 111 patients was 20.2 and 22.4 months (2.7‐50.0) for the entire cohort and surviving patients.

Most patients (85.6%) present with local advanced disease (T3‐4) including 11 patients with neck adenopathy. ACC (35.1%), sarcoma (20.7%), squamous cell carcinoma (17.1%), and melanoma (9.9%) constituted the majority of histology subtypes. Approximately 65% of the cohort received surgery. Thirty patients had biopsy only, and 9 had no surgery/biopsy for their locally recurrent disease. Ninety‐two patients (82.9%) had gross tumor prior to PBRT. Thirty patients received particle‐beam radiation therapy (PBRT) as re‐irradiation for salvage treatment. Nineteen (17.1%) patients received concurrent chemotherapy. The characteristics of the patients were detailed in Table 1.

**TABLE 1:** Characteristics of patients

Characteristics	N (%)
Total	111 (100)
Gender	
Male	64 (57.7)
Female	47 (42.3)
Age	
Median (range), y	49 (14‐85)
Histopathology	
ACC	39 (35.1)
Sarcoma	23 (20.7)
SCC	19 (17.1)
Melanoma	11 (9.9)
ONB	9 (8.1)
Adenocarcinoma	3 (2.7)
Myoepithelial carcinoma	2 (1.8)
Mucoepidermoid carcinoma	1 (0.9)
Poor‐differentiated carcinoma	1 (0.9)
Neuroendocrine carcinoma	1 (0.9)
Salivary origin malignancy	1 (0.9)
SMARCB1 (INI‐1)‐deficient carcinoma	1 (0.9)
Radiation‐induced second primary malignancies	
Yes	4 (3.6)
No	107 (96.4)
T category	
T1	7 (6.3)
T2	9 (8.1)
T3	18 (16.2)
T4	77 (69.4)
N category	
N0	100 (90.1)
N1‐3	11 (9.9)
Surgery	
Without surgery	9 (8.1)
With surgery	72 (64.9)
Biopsy	30 (27.0)
Surgical margin*	
R1/R0	19 (17.1)
R2/Biopsy	92 (82.9)
Gross tumor	
With gross tumor	92 (82.9)
Without gross tumor	19 (17.1)
GTV ml, median (range)	51 (0‐225.6)
Tumor status	
Primary	76 (68.5)
Recurrence	35 (31.5)
Re‐irradiation	
Yes	30 (27.0)
No	81 (73.0)
PRT dose	
56 Gy (RBE)/28 frs.	2 (1.8)
66 Gy (RBE)/33 frs.	1 (0.9)
70 Gy (RBE)/35 frs.	1 (0.9)
CIRT dose	
60 Gy (RBE)/20 frs.	4 (3.6)
63 Gy (RBE)/18 frs.	22 (19.8)
63 Gy (RBE)/21 frs.	26 (23.4)
66 Gy (RBE)/22 frs.	6 (5.4)
70 Gy (RBE)/20 frs.	10 (9.0)
73.5 Gy (RBE)/21 frs.	2 (1.8)
PRT + CIRT dose	
69 Gy (RBE)/32 frs.	2 (1.8)
71 Gy (RBE)/33 frs.	24 (21.6)
73.5 Gy (RBE)/33 frs.	11 (9.9)
Radiation technique	
PRT	4 (3.6)
CIRT	70 (63.1)
PRT and CIRT	37 (33.3)
Concurrent chemotherapy	
Yes	19 (17.1)
No	92 (82.9)

Abbreviations: ACC, adenoid cystic carcinoma; CIRT, carbon‐ion radiotherapy; ONB, olfactory neuroblastoma; PRT, proton radiotherapy; SCC, squamous cell carcinoma.

* Surgical margin was not reported for some of the patients. Those patients were grouped to R1/R0 or R2/biopsy according to MRI findings.

### 3.2 Particle‐beam radiation therapy

All of patients completed planned PBRT without break. Elective nodal irradiation was performed in 40 patients, according to the neck lymph nodes status, tumor histology, and stage.

Two patients with T4N0M0 ACC of oral cavity and maxillary sinus achieved R0 resection received PRT to 56 Gy (RBE) in 28 daily fractions. Two patients with T3N0M0 SCC of maxillary sinus and T2N0M0 ACC of nasal cavity received PRT to 70 and 66 Gy (RBE) in 35 and 30 fractions, respectively. Four patients achieved R0 resection of SCC or adenocarcinoma received CIRT to 60 Gy (RBE) in 20 fractions. The remaining 66 patients with gross disease or R1 resection who received CIRT to 63‐73.5 Gy (RBE) in 18‐21 fractions to the CTV‐G for gross tumors and surgical bed, and 54‐63 Gy (RBE) in 18‐21 fractions for CTVs of the high‐risk region using simultaneous integrated boost technique. Thirty‐seven patients received a combination of PRT and CIRT boost were treated to 54‐56 Gy (RBE) in 27‐30 fractions using PRT to the CTVs of the high‐risk region, followed by CIRT boost to 15‐17.5 Gy (RBE) in 5 fractions to the CTV‐G or CTV‐N.

### 3.3 Disease control and survival outcomes

**FIGURE 1:**
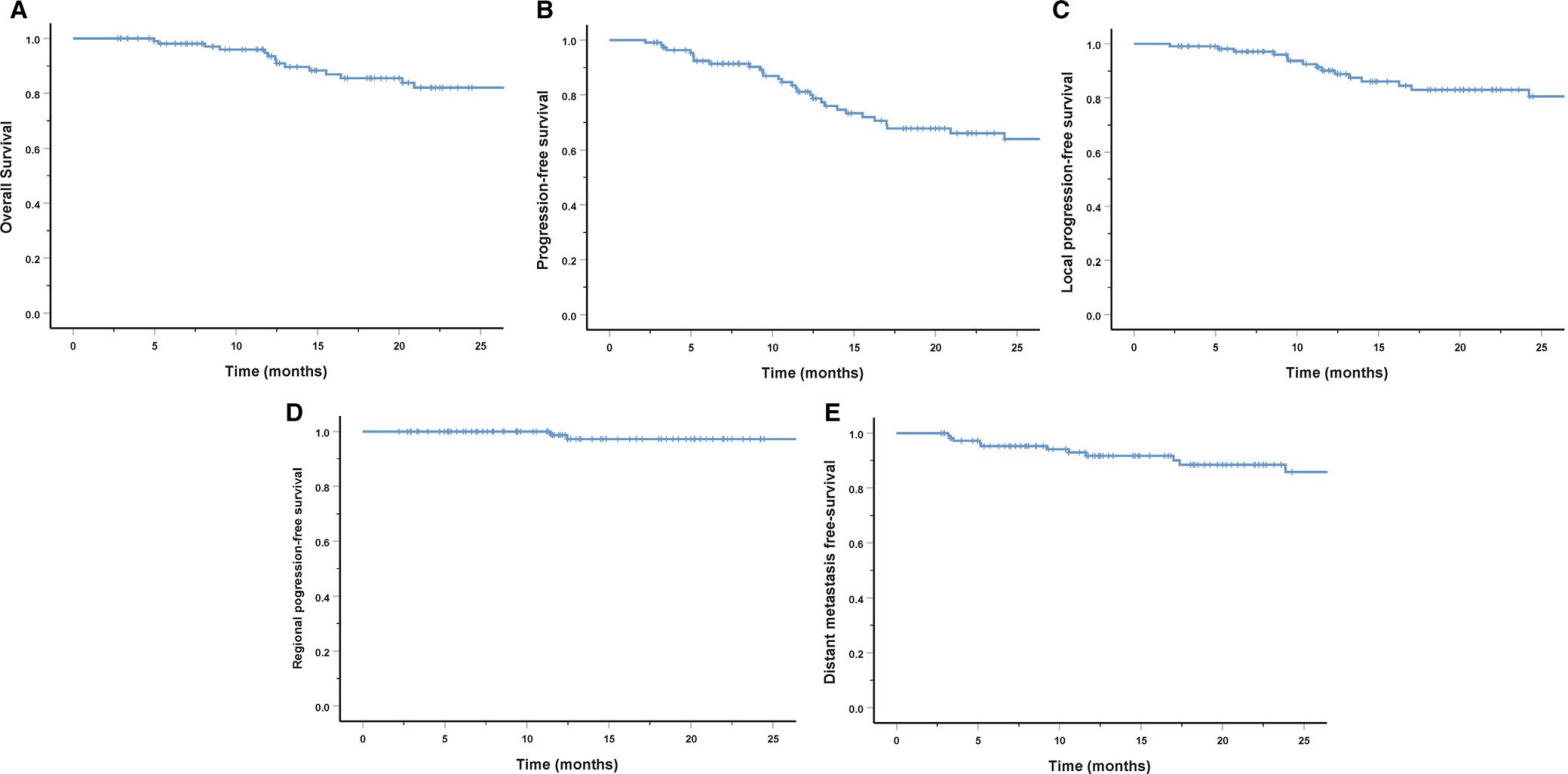
Two‐year survival rates for entire cohorts: overall survival (A), progression‐free survival (B), local progression‐free survival (C), regional progression‐free survival (D), and distant metastasis‐free survival (E)

With a median follow‐up time of 20.2 (range 2.7‐50.0) months, in all cohort, 15 patients (13.5%) developed local failure including 8 in‐field, 2 marginal, 2 out‐field, and 3 cross‐compartment recurrences. Additionally, 2 patients developed neck node recurrence only. DM was observed in 13 (11.7%) patients. Seventeen patients (15.3%) died including 3 from unrelated conditions. The 2‐year LPFS, RPFS, DMFS, PFS, and OS rates were 83%, 97.2%, 85.9%, 66%, and 82%, respectively. Those rates were detailed in Figure 1.

For the 81 patients who received naive‐RT, local and regional recurrent occurred in 9 (11.1%) and 2 (2.5%) of them, 12 patients (14.8%) developed DM and 11 patients (13.6%) died including 1 from unrelated condition. The 2‐year LPFS, RPFS, DMFS, PFS, and OS rates were 85.6%, 96.2%, 82.2%, 65.5%, and 83.7%, respectively.

### 3.4 Prognosis factors

Both uni‐ and multivariate analyses (UVA and MVA) were used to assessed potential predictive factors for survivals. Surgical margin status was excluded in both UVA and MVA due to the small number of patients (6 patients) who achieved R0 resection, and status on surgical margin was missing for some of the patients, it was not included in the analysis.

**FIGURE 2:**
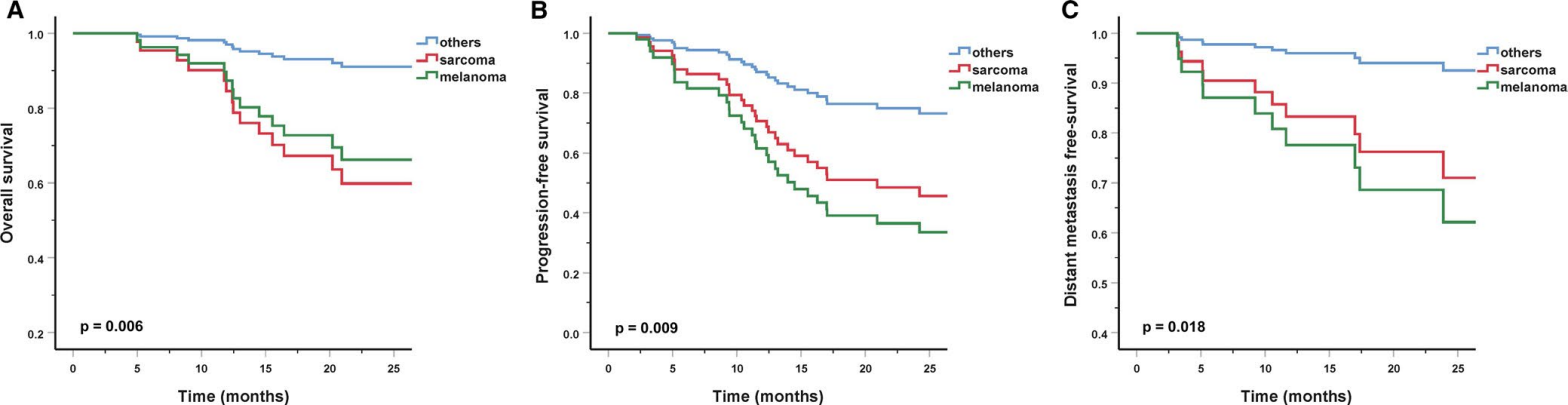
Overall survival (A), progression‐free survival (B), and distant metastasis‐free survival (C) with sarcoma vs melanoma vs other histologies

In UVA, for the entire cohort, in addition to gender, patients with both melanoma and sarcoma had worse OS, DMFS, and PFS. Patients with T1/2 disease had worse OS and PFS as compared to those with T3/4 diseases, as 66.7% of the T1/2 patients had sarcoma. As expected, patients with N+ disease had significantly worse PFS. Moreover, larger GTV increased the risk of distant metastasis (Table 2; Figure 2). A similar situation was observed in RT‐naive patients, the histological type, T and N categories were significantly associated with their survivals. And a higher BED had a trend to improved OS and PFS in RT‐naive patients when BED was taken as a continuous variable (Table 3).

**TABLE 2:** Univariate analysis of survival rates for entire cohort by Cox regression

Variables	OS		PFS		LPFS		DMFS	
	HR (95% CI)	*P* value	HR (95% CI)	*P* value	HR (95% CI)	*P* value	HR (95% CI)	*P* value
Gender (male as ref.)	0.22 (0.06‐0.78)	**.019**	0.40 (0.19‐0.83)	**.015**	0.21 (0.06‐0.75)	**.016**	0.66 (0.21‐2.01)	.46
Age (≤49 y vs >49 y)	2.06 (0.78‐5.46)	.15	1.29 (0.66‐2.53)	.46	1.76 (0.66‐4.74)	.26	0.81 (0.26‐2.50)	.72
Histology (others as ref.)								
Sarcoma	5.49 (1.87‐16.08)	**.02**	2.51 (1.16‐5.45)	.20	1.2 (0.39‐3.74)	.75	4.42 (1.28‐15.3)	**.019**
Melanoma	4.4 (1.09‐17.8)	**.038**	3.5 (1.37‐9.0)	**.009**	0	.98	6.14 (1.42‐26.5)	**.015**
T categories (T1/2 vs T3/4)	0.37 (0.14‐0.99)	**.047**	0.44 (0.21‐0.95)	**.036**	0.59 (0.19‐1.84)	.36	0.38 (0.12‐1.22)	.10
N categories (N0 vs N1‐3)	2.72 (0.87‐8.46)	.09	2.86 (1.23‐6.66)	**.015**	1.17 (0.27‐5.18)	.83	3.07 (0.84‐11.24)	.09
GTV (continuous variable)	1.01 (1.0‐1.02)	.14	1.00 (1.0‐1.01)	.21	1.0 (0.99‐1.01)	.72	1.01 (1.0‐1.02)	**.028**
Tumor status (primary vs recurrence)	1.7 (0.65‐4.46)	.27	1.29 (0.65‐2.59)	.47	1.46 (0.54‐3.92)	.46	0.93 (0.29‐3.04)	.91
Radiotherapy (RT‐naive vs re‐irradiation)	1.71 (0.61‐4.78)	.31	1.18 (0.56‐2.47)	.66	2.14 (0.80‐5.75)	.13	0.23 (0.03‐1.77)	.16
Surgery (surgery vs without surgery + biopsy)	0.92 (0.34‐2.49)	.92	0.98 (0.48‐1.99)	.96	1.07 (0.39‐2.95)	.90	1.09 (0.36‐3.35)	.88
BED (continuous variable)	1.03 (0.93‐1.13)	.64	1.01 (0.94‐1.08)	.79	0.97 (0.89‐1.05)	.44	1.07 (0.95‐1.21)	.26

Abbreviations: BED, biological equivalent dose; DMFS, distant metastasis‐free survival; HR, Hazard ratio; LPFS, local progression‐free survival; OS, overall survival; PFS, progression‐free survival.

Statistically significant differences indicated in bold type.

**TABLE 3:** Univariate analysis of survival rates for naive‐RT patients by Cox regression

Variables	OS		PFS		LPFS		DMFS	
	HR (95% CI)	*P* value	HR (95% CI)	*P* value	HR (95% CI)	*P* value	HR (95% CI)	*P* value
Gender (male as ref.)	0.4 (0.11‐1.55)	.19	0.47 (0.2‐1.1)	.08	0.27 (0.06‐1.3)	1.0	0.52 (0.16‐1.73)	.29
Age (≤49 y vs >49 y)	0.96 (0.28‐3.31	.95	1.14 (0.5‐2.56)	.76	1.36 (0.36‐5.05)	.65	1.16 (0.37‐3.69)	.80
Histology (others as ref.)								
Sarcoma	12.73 (2.5‐66.27)	**.003**	2.7 (1.04‐6.96)	**.041**	0.98 (2.0‐4.73)	.98	3.18 (0.85‐11.9)	.09
Melanoma	8.93 (1.48‐53.71)	**.017**	3.9 (1.42‐10.78)	**.008**	0	.99	4.38 (1.04‐18.9)	**.048**
T categories (T1/2 vs T3/4)	0.3 (0.88‐1.03)	**.056**	0.38 (0.15‐0.96)	**.041**	0.6 (0.12‐2.9)	.53	0.42 (0.11‐1.55)	.19
N categories (N0 vs N1‐3)	2.63 (0.68‐10.2)	.16	2.75 (1.08‐7.02)	**.035**	0.76 (0.1‐6.08)	.80	2.65 (0.71‐9.88)	.15
GTV (continuous variable)	1.01 (1.0‐1.02)	**.06**	1.01 (1.0‐1.01)	.09	1.0 (1.0‐1.02)	.54	1.01 (1.0‐1.02)	.1
Surgery (surgery vs without surgery + biopsy)	1.55 (0.41‐5.89)	.52	1.1 (0.47‐2.58)	.82	1.015 (0.29‐4.6)	.84	0.78 (0.25‐2.45)	.67
BED (continuous variable)	1.19 (1.01‐1.39)	**.033**	1.14 (1.02‐1.27)	**.019**	1.13 (0.94‐1.35)	.21	1.09 (0.94‐1.27)	.26

Abbreviations: BED, biological equivalent dose; DMFS, distant metastasis‐free survival; HR, Hazard ratio; LPFS, local progression‐free survival; OS, overall survival; PFS, progression‐free survival.

Statistically significant differences indicated in bold type.

**FIGURE 3:**
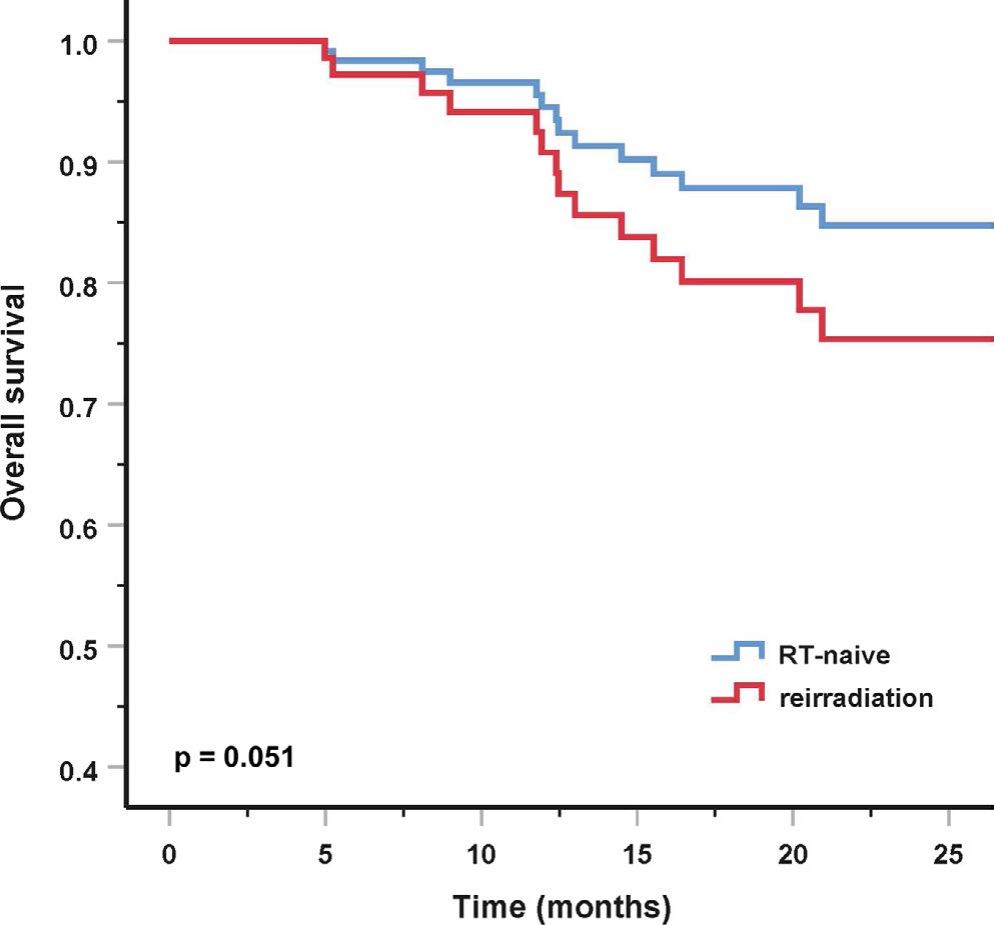
Overall survival with radiation therapy (RT)‐naive vs re‐irradiation

In MVA, for the entire cohort, tumor volume instead of T‐category was used because the variation of the TNM staging system for different histologies (such as sarcoma). A larger GTV had significantly worse DMFS and OS. Melanoma and sarcoma patients had significantly higher distant metastatic rate, and poorer OS and PFS. Male had poorer survivals as compared with female. Furthermore, patients who underwent re‐irradiation had significantly worse OS (Table 4; Figure 3). For RT‐naive patients, histological type was also significantly associated with OS, PFS, and DMFS. And a larger GTV had significantly worse DMFS and OS. Not only that, a higher BED was also found to improve OS in RT‐naive patients (Table 5).

**TABLE 4:** Multivariate analysis of survival rates for entire cohort by Cox regression

Variables	OS		PFS		LPFS		DMFS	
	HR (95% CI)	*P* value	HR (95% CI)	*P* value	HR (95% CI)	*P* value	HR (95% CI)	*P* value
Gender (male as ref.)	0.15 (0.04‐0.59)	**.006**	0.33 (0.15‐0.71)	**.005**	0.2 (0.55‐0.73)	**.014**	0.66 (0.21‐2.01)	.46
Age (≤49 y vs >49 y)	2.91 (0.91‐9.33)	.07	1.3 (0.64‐2.69)	.49	1.78 (0.61‐5.26)	.29	0.91 (0.27‐3.05)	.87
Histology (others as ref.)								
Sarcoma	9.3 (2.56‐33.75)	**.001**	2.82 (1.21‐6.56)	**.016**	1.71 (0.51‐5.73)	.39	5.01 (1.25‐20.1)	**.02**
Melanoma	17.4 (3.1‐97.39)	**.001**	6.3 (2.2‐18.07)	**.001**	0	.98	8.49 (1.61‐44.6)	**.01**
N categories (N0 vs N1‐3)	1.63 (0.48‐5.58)	.44	1.62 (0.58‐4.48)	.36	0.88 (0.16‐4.97)	.89	1.62 (0.4‐6.6)	.5
GTV (continuous variable)	1.0 (0.25‐4.06)	**.058**	1.0 (1.0‐1.02)	.13	1.0 (0.98‐1.01)	.97	1.01 (1.0‐1.03)	**.03**
Radiotherapy (RT‐naive vs re‐irradiation)	5.69 (1.0‐35.51)	**.051**	1.96 (0.68‐5.63)	.21	1.79 (0.48‐6.61)	.38	0.48 (0.04‐5.8)	.57
Surgery (surgery vs without surgery + biopsy)	0.37 (0.1‐1.41)	.15	0.75 (0.34‐1.64)	.47	0.9 (0.3‐2.7)	.85	1.1 (0.3‐4.03)	.89
BED (continuous variable)	1.07 (0.93‐1.22)	.37	1.0 (0.93‐1.1)	.84	1.0 (0.89‐1.13)	.92	1.0 (0.86‐1.13)	.82

Abbreviations: BED, biological equivalent dose; DMFS, distant metastasis‐free survival; HR, Hazard ratio; LPFS, local progression‐free survival; OS, overall survival; PFS, progression‐free survival.

Statistically significant differences indicated in bold type.

**TABLE 5:** Multivariate analysis of survival rates for naive‐RT patients by Cox regression

Variables	OS		PFS		LPFS		DMFS	
	HR (95% CI)	*P* value	HR (95% CI)	*P* value	HR (95% CI)	*P* value	HR (95% CI)	*P* value
Gender (male as ref.)	0.4 (0.11‐1.55)	.19	0.47 (0.2‐1.1)	.08	0.27 (0.06‐1.3)	1.0	0.52 (0.16‐1.73)	.29
Age (≤49 y vs >49 y)	0.96 (0.28‐3.31	.95	1.14 (0.5‐2.56)	.76	1.36 (0.36‐5.05)	.65	1.16 (0.37‐3.69)	.80
Histology (others as ref.)								
Sarcoma	12.73 (2.5‐66.27)	**.003**	2.7 (1.04‐6.96)	**.041**	0.98 (2.0‐4.73)	.98	3.18 (0.85‐11.9)	.09
Melanoma	8.93 (1.48‐53.71)	**.017**	3.9 (1.42‐10.78)	**.008**	0	.99	4.38 (1.04‐18.9)	**.048**
T categories (T1/2 vs T3/4)	0.3 (0.88‐1.03)	**.056**	0.38 (0.15‐0.96)	**.041**	0.6 (0.12‐2.9)	.53	0.42 (0.11‐1.55)	.19
N categories (N0 vs N1‐3)	2.63 (0.68‐10.2)	.16	2.75 (1.08‐7.02)	**.035**	0.76 (0.1‐6.08)	.80	2.65 (0.71‐9.88)	.15
GTV (continuous variable)	1.01 (1.0‐1.02)	**.06**	1.01 (1.0‐1.01)	.09	1.0 (1.0‐1.02)	.54	1.01 (1.0‐1.02)	.1
Surgery (surgery vs without surgery + biopsy)	1.55 (0.41‐5.89)	.52	1.1 (0.47‐2.58)	.82	1.015 (0.29‐4.6)	.84	0.78 (0.25‐2.45)	.67
BED (continuous variable)	1.19 (1.01‐1.39)	**.033**	1.14 (1.02‐1.27)	**.019**	1.13 (0.94‐1.35)	.21	1.09 (0.94‐1.27)	.26

Abbreviations: BED, biological equivalent dose; DMFS, distant metastasis‐free survival; HR, Hazard ratio; LPFS, local progression‐free survival; OS, overall survival; PFS, progression‐free survival.

Statistically significant differences indicated in bold type.

### 3.5 Toxicities

Seventy‐nine (71.2%) patients experienced grade 1 or 2 acute toxicities included dermatitis and mucositis, xerostomia, facial edema, tinnitus, visual decreased, epiphora, diplopia, conjunctival congestion, and/or facial numbness. No acute toxicities of grade 3 or above were observed.

Twenty‐two (19.8%) patients experienced grades 1‐2 late toxicities including xerostomia, facial edema, tinnitus, visual decreased, epiphora, diplopia, blepharoptosis, and brain injury. Only 4 (3.6%) patients developed grades 3‐4 late toxicities (Table 6). Two patients developed ipsilateral vision impairment: 1 with T4 tumor with orbital and orbital apex invasion developed grade 3 vision impairment at 2 years after PBRT to 69 Gy (RBE) in 32 daily fractions (PRT: 54 Gy (RBE) in 27 daily fractions, followed by CIRT boost: 15 Gy (RBE) in 5 fractions); another with orbital invasion developed grade 4 ipsilateral vision impairment after CIRT re‐irradiation to 63 Gy (RBE) in 18 daily fractions. One osteosarcoma patient developed osteonecrosis after CIRT re‐irradiation to 63 Gy (RBE) in 21 daily fractions and was salvaged with debridement. The patient is alive without evidence of disease at her last follow‐up. One additional patient who received PRT to 66 Gy (RBE) in 30 fractions gradually progress to grade 3 xerostomia after completion of treatment.

**TABLE 6:** Characteristics of late toxicities

Type of adverse reaction	Late toxicities			
	Grade 1, N (%)	Grade 2, N (%)	Grade 3, N (%)	Grade 4, N (%)
Facial edema	1 (0.9)	0	0	0
Decreased vision	1 (0.9)	0	1 (0.9)	1 (0.9)
Epiphora	1 (0.9)	0	0	0
Diplopia	1 (0.9)	0	0	0
Blepharoptosis	1 (0.9)	0	0	0
Abducent restriction	1 (0.9)	0	0	0
Tinnitus	2 (1.8)	0	0	0
Hearing decrease	1 (0.9)	0	0	0
Xerostomia	8 (7.2)	5 (4.5)	1 (0.9)	0
Osteonecrosis	0	0	1 (0.9)	0
Brain injury	1 (0.9)	0	0	0

## 4 DISCUSSION

Our retrospective analysis of 111 patients with SNM treated with intensity‐modulated PBRT revealed favorable survivals and toxicity profile. With a median follow‐up time of 20.2 months, the 2‐year OS, PFS, LPFS, RPFS, and DMFS rates were 82.0%, 66.0%, 83.0%, 97.2%, and 85.9% for entire cohort, and 85.6%, 96.2%, 82.2%, 65.5%, and 83.7% for RT‐naive patients. Sarcoma and melanoma patients had significantly poorer outcomes as compared with other histologic types. Patients with lager tumor volume suffered from a worse OS and DMFS. Not surprisingly, the survival outcomes of patients received salvage re‐irradiation had a poorer OS compared with RT‐naive patients. In addition, no patient suffered from severe (ie, grades 3~5) acute toxicity, and severe late toxicities was observed in only 4 patients.

Although surgery is the mainstay approach for the management of SNM, local control (LC) after surgery alone is merely 40%~50%.^2^, ^18^, ^19^ Adjuvant RT is usually needed for patients with close or positive resection margins. For patients with unresectable or incompletely resected disease, radiotherapy is the only definitive treatment modality. With the prevailing use of IMRT, the 2‐year LC and disease‐free survival (DFS) rates approached 62%~76% and 60%, substantially improved from those after conventional radiation technology.^5^, ^6^, ^7^, ^8^, ^9^ The effectiveness and toxicity profile of PBRT has been studied in a few retrospective studies. In addition, outcomes of PBRT and photon‐based RT were compared in a well‐conducted meta‐analysis which included 286 particle beam irradiated and 1186 photon beam irradiated patients. Patients received PBRT (proton = 238, CIRT = 58) were compared to those received photon‐based 2‐dimentional (2D), 3D conformal, and IMRT. The use of PBRT provided better locoregional control, OS, as compared to photon‐treated patients, and also found that proton therapy improved DFS as compared to IMRT.^14^

Dagan et al reported clinical outcomes of RT‐naive SNM patients (excluding sarcoma and melanoma) treated with proton therapy. Their 3‐year LC and OS were 83% and 68%, respectively. Patients with gross residual disease suffered from worse LC significantly.^20^ More recently, researchers from the Memorial Sloan Kettering Cancer Center demonstrated favorable outcomes from their 86 SNM patients (excluding sarcoma) treated with proton therapy. With a median follow‐up time of 23.4 months, the 2 years LC and OS rates were 83% and 81% for RT‐naive patients, and 77% and 66% for re‐irradiation patients. Both UVA and MVA revealed that re‐irradiation patients had a worse OS than RT‐naive patients.^21^ Our data were comparable with those of the current study, although more heterogeneous histologies and more patients with T3/T4/N+ disease were included in our series. Our 2‐year OS and LPFS were 82% and 83%, respectively. We also found that patients had a significantly worse OS after re‐irradiation using CIRT alone. In our previous study, we demonstrated that salvage using CIRT is feasible and safe for locally recurrent head and neck cancer patients. The 1‐year OS of 96% after salvage CIRT was achieved.^22^

Not surprisingly, our data revealed that larger GTV associated with higher DM rate, and poor OS. The management of locally advanced malignancies of the sinonasal track has always been challenging due to their complex anatomic background. In a retrospective study of 39 unresectable SNM patients (excluding sarcoma) with N0M0 disease treated with proton therapy, the 3‐year PFS and OS rates of 49.1% and 59.3% were reported, respectively.^23^ Another study on locally advanced N0M0 SNM (47 after PRT and 10 after CIRT, sarcoma excluded) patients, 3‐year LPFS, and OS rates of 56% and 61% were achieved after proton therapy, respectively.^24^ In a more recent publication, CIRT produced 2‐year LC and OS rates of 84.1%, and 79.6%, respectively, for RT‐naive patients with locally advanced SNM. The study also showed that both larger GTV and melanoma histology were associated with worse OS.^15^ In our cohort, 85.6% of the patients had T3/4 disease, and 61% of them received CIRT only for treatment. The 2‐year LPFS and OS of these patients were 85.2% and 86.7%, respectively. It is difficult to compare our outcomes directly with published data due to the substantial differences in pathology type. Approximately 20% and 10% of our patients had sarcoma and melanoma. Both UVA and MVA for our data indicated that sarcomas and melanoma had a worse OS, PFS, and DMFS as compared with other histological type.

Historically, acute mucositis and dermatitis close to 50%~80% were observed after IMRT, and the severity of 15%~37% of patients were grade 3 or higher.^6^, ^7^, ^8^ The grade 3 or 4 acute ocular toxicities was reportedly 5%, but over 10% patients experienced grade ≥3 late ocular impairment after IMRT.^6^, ^7^, ^8^ The unique physical characteristic of Bragg Peak of PBRT allows a high‐dose coverage to tumor with low entrance and exit dose.^25^, ^26^ In addition, the RBE of PBRT increases with increasing LET and the depth of particle beam. The synergy of both above‐mentioned physical properties of PBRT made it an optimal technology for deep‐seated lesions close to vital OARs. Therefore, the dose distribution of PBRT including PRT and CIRT improved dramatically as compared with IMRT.^10^, ^11^, ^12^ The reported acute grade mucositis and dermatitis ranged between 0%~19% and 0%~7%, respectively, after PBRT. And PBRT‐induced severe (grade 3 or 4) late toxicity such as vision decrease, brain necrosis, osteoradionecrosis, or neuropathy ranged between 1%~3% overall.^12^, ^13^, ^15^, ^20^, ^23^, ^27^ However, for patients with locally advanced disease treated with PBRT, the incidence of severe late toxicities rate would increase to 5%‐23%.^15^ In our study, PBS technology was used and might be the underlying reason for a lower toxicity rate.^28^, ^29^, ^30^ No patient experienced grade 3 or 4 acute. There was no grade 3 or above acute toxicity observed. In the 4 (3.6%) patients who experienced grades 3‐4 late toxicities, 2 developed ipsilateral vision impairment: 1 patient with T4 disease with orbital invasion developed grade 3 vision impairment in 2 years after completion of PRT combined CIRT; Another patient with recurrence and orbital invasion received CIRT re‐irradiation then experienced grade 4 ipsilateral vision impairment. Osteonecrosis was observed in 1 patient with osteosarcoma, and the patient received debridement and live without disease at last follow up.

A number of pitfalls need to be discussed for our study. First, although all patients were treated according to either clinical trial or our institutional standard operative protocol designed for sinonasal malignancies, patients included in this analysis are of heterogenous histological subtypes and their PBRT technology used was mixed. The retrospective nature of this analysis suffered from selection bias. Second, a follow‐up time of close to 2 years is relatively short for this group of patients considering their relatively good OS data. Despite of our relatively favorable survival outcomes demonstrated and superb toxicity profile, longer follow‐up will be needed to understand the longer‐term survival outcomes as well as late toxicities for this group of patients.

Future investigations preferably in prospective randomized fashion will be needed to confirm the benefit of CIRT for patients with sinonasal malignancies. Furthermore, due to the poorer prognosis in patients with sarcoma and malignant melanoma, different histological subtypes should be investigated in separate clinical trials. Due to the rarity of the disease, international collaboration is necessary for multi‐institutional randomized clinical trials.

## 5 CONCLUSION

The current study showed that PBRT is an effective and safe treatment option for patients with SNM. Both 2‐year overall and local progression‐free survival rates exceeded 80% for our patients largely with locally advanced and recurrent diseases. PBRT‐induced acute and late toxicities were both infrequent and not severe. Only 4 patients experienced grade 3 late toxicities. Further follow‐up was necessary to confirm the long‐term benefit of PBRT for patients with SNM.

## CONFLICT OF INTERESTS

None.

## AUTHORS' CONTRIBUTIONS

Weixu Hu: design study, collect data and assembly, data analysis, interpretation, drafting, revising article, final approval. Jiyi Hu: collect data, data analysis, drafting, revision, final approval. Jing Gao: collect data, drafting, revision, approve of the version. Jing Yang: collect data, revision, manuscript final approval. Xianxin Qiu: data acquisition, revision, final version approval. Lin Kong: Conceptualization and design study, supervision, data analysis, results interpretation, drafting and revising article, funding acquisition, final approval. Jiade J. Lu: conceptualization and design study, supervision, data analysis, results interpretation, drafting and revising the article, language editing, final approval.

## Data Availability

The data that support the findings of this study are available from the corresponding author upon reasonable request.
